# Trends in nephrology referral patterns for patients with chronic kidney disease: Retrospective cohort study

**DOI:** 10.1371/journal.pone.0272689

**Published:** 2022-08-11

**Authors:** Anukul Ghimire, Feng Ye, Brenda Hemmelgarn, Deenaz Zaidi, Kailash K. Jindal, Marcello A. Tonelli, Matthew Cooper, Matthew T. James, Maryam Khan, Mohammed M. Tinwala, Naima Sultana, Paul E. Ronksley, Shezel Muneer, Scott Klarenbach, Ikechi G. Okpechi, Aminu K. Bello

**Affiliations:** 1 Division of Nephrology, Faculty of Medicine and Dentistry, University of Alberta, Edmonton, Alberta, Canada; 2 Department of Medicine, Cumming School of Medicine, University of Calgary, Calgary, Alberta, Canada; 3 Department of Community Health Sciences, Cumming School of Medicine, University of Calgary, Calgary, Alberta, Canada; Postgraduate Medical Institute, INDIA

## Abstract

**Introduction:**

Information on early, guideline discordant referrals in nephrology is limited. Our objective was to investigate trends in referral patterns to nephrology for patients with chronic kidney disease (CKD).

**Methods:**

Retrospective cohort study of adults with ≥1 visits to a nephrologist from primary care with ≥1 serum creatinine and/or urine protein measurement <180 days before index nephrology visit, from 2006 and 2019 in Alberta, Canada. Guideline discordant referrals were those that did not meet ≥1 of: Estimated glomerular filtration rate (eGFR) ˂ 30 mL/min/1.73m^2^, persistent albuminuria (ACR ≥ 300 mg/g, PCR ≥ 500 mg/g, or Udip ≥ 2+), or progressive and persistent decline in eGFR until index nephrology visit (≥ 5 mL/min/1.73m^2^).

**Results:**

Of 69,372 patients with CKD, 28,518 (41%) were referred in a guideline concordant manner. The overall rate of first outpatient visits to nephrology increased from 2006 to 2019, although guideline discordant referrals showed a greater increase (trend 21.9 per million population/year, 95% confidence interval 4.3, 39.4) versus guideline concordant referrals (trend 12.4 per million population/year, 95% confidence interval 5.7, 19.0). The guideline concordant cohort were more likely to be on renin-angiotensin system blockers or beta blockers (hazard ratio 1.14, 95% confidence interval 1.12, 1.16), and had a higher risk of CKD progression (hazard ratio 1.09, 95% confidence interval 1.06, 1.13), kidney failure (hazard ratio 7.65, 95% confidence interval 6.83, 8.56), cardiovascular event (hazard ratio 1.40, 95% confidence interval 1.35,1.45) and mortality (hazard ratio 1.58, 95% confidence interval 1.52, 1.63).

**Conclusions:**

A significant proportion nephrology referrals from primary care were not consistent with current guideline-recommended criteria for referral. Further work is needed to identify quality improvement initiatives aimed at enhancing referral patterns of patients with CKD.

## Introduction

It is estimated that the number of patients with kidney failure requiring kidney replacement therapy (KRT) will increase worldwide to 5.439 million by 2030 [1]. Management of chronic kidney disease (CKD) in early stages is important to prevent progression to kidney failure [2], and primary care providers (PCPs) play an essential role in this process.

Kidney Disease: Improving Global Outcomes (KDIGO) has published recommendations based on laboratory and clinical data regarding the timing of specialist referrals for patients with CKD [3]. Timely referral to a nephrologist has been shown to be associated with initiation of appropriate pharmacological therapies, increased utilization of peritoneal dialysis, and reduction in mortality [4, 5]. However, a significant number of nephrology referrals are not concordant with current guidelines, and it may be more appropriate for those patients to by managed by PCPs rather than specialists [6, 7].

Late referrals to nephrology have been well-studied, and can lead to increased hospitalization, higher costs, increased mortality, and worse outcomes after initiation of dialysis [8–11]. However, evidence is scant for the opposite end of the referral spectrum (i.e., patients who may not require specialist care). The guideline discordant (GD) referrals are important, as they undoubtedly lead to increased costs and longer wait times. We assessed referral patterns to nephrology in Alberta, Canada from 2006 to 2019 and evaluated differences in characteristics and outcomes between patients referred in guideline concordant (GC) and GD ways.

## Material and methods

### Study population and data sources

The study cohort comprised patients over age 18 in Alberta, Canada who had their first outpatient visit to a nephrologist between April 1, 2005 and March 31, 2019. We obtained data from the Alberta Kidney Disease Network (AKDN) database, which incorporates data from Alberta Health such as provincial health registry status, physician claims, hospital discharge abstracts, and ambulatory care utilization; the Northern and Southern Alberta Renal Programs to capture patients receiving chronic dialysis; and clinical laboratories in Alberta [12]. We obtained drug prescription information from the Pharmaceutical Information Network (PIN) database. To be eligible for inclusion, patients must have had at least 1 outpatient serum creatinine (SCr) or albuminuria measurement within the 180 days prior to the initial nephrology visit. We excluded participants who had progressed to kidney failure and had received chronic dialysis or a kidney transplant before the initial outpatient nephrology visit. We assigned an index date for all participants in the study, corresponding to the date of their first outpatient visit to a nephrologist. The study was approved by health research ethics boards at the Universities of Alberta and Calgary and participant consent was not required by the ethics boards given that the data was de-identified.

### Assessment of baseline kidney function and albuminuria

We used the Chronic Kidney Disease Epidemiology Collaboration (CKD-EPI) equation to calculate eGFR [13]. We estimated baseline kidney function using the most recent outpatient SCr measurement within the 180 days prior to the initial nephrology visit. We delineated three categories for baseline eGFR: ≥ 60 mL/min/1.73m^2^, 15–59 mL/min/1.73m^2^, and ≤ 15 mL/min/1.73m^2^ [3]. We ascertained albuminuria from all outpatient measurements of albumin-creatinine ratio (ACR) and protein-creatinine ratio (PCR), as well as urine dipstick (Udip) tests during the same period. Albuminuria was defined as ACR ≥ 300 mg/g, PCR ≥ 500 mg/g, or Udip ≥ 2+ [3].

### Evaluation of referrals to nephrology

We defined a referral as GC based on eGFR category, decline in eGFR, and albuminuria during the six months prior to the initial nephrology visit. Our criteria reflect KDIGO guidelines, which are based on evidence of the benefits of timely referral, including delaying the need to initiate KRT and improved survival [3]. Our specific criteria were:

- A most recent eGFR < 30 mL/min/1.73m^2^, or
- Presence of persistent albuminuria on at least two consecutive measurements prior to the index nephrology visit, or
- Progressive decline in eGFR (≥ 5 mL/min/1.73m^2^ decrease from the first eGFR measurement that persisted until the index nephology visit).

GC referrals met any of the above criteria, whereas GD referrals did not meet any of the above criteria. We classified participants without an eGFR or albuminuria measurement in a separate category.

### Assessment of demographic characteristics and co-morbid conditions

We recorded baseline demographic data, including age, sex, and postal code of residence from the Alberta Health administrative data files. We linked postal codes to the Canadian Census using the Postal Code Conversion File (www.statcan.ca) to determine rural versus urban residential location. We assessed material deprivation based on the Pampalon Deprivation Index (1 = least deprived to 5 = most deprived) [14, 15]. We used ArcInfo software (version 10.0, ESRI) to determine the shortest distance by road between each patient’s residence and the nearest nephrologist, as previously described [16, 17]. We delineated three categories for distance: < 50 km, 50–100 km, and >100 km. Data were complete except for material deprivation index and distance (1.8% and <1% missing); affected participants were assigned to a missing data category.

We identified pre-existing co-morbid conditions from hospital discharge records, physician claims, and ambulatory care classification system files based on validated algorithms [18, 19]. We categorized urine red blood cell count (URBC) data from provincial laboratories as negative, trace, small, moderate, or large, and defined hematuria as persistent URBC > trace in the six months prior to the first nephrologist visit.

### Study outcomes

The primary outcome was the annual rate of initial outpatient visits to nephrologists, expressed as a number per million population (pmp). The population count was obtained from the Alberta Health provincial health registry file [12]. We calculated the annual rate of nephrology visits and estimated the secular trend overall and in each referral group (GC and GD) from 2006 to 2019.

Secondary outcomes included annual rates of initial outpatient visits to other internal medicine specialists (general internal medicine, cardiology, and endocrinology). We also studied the associations between referral characteristics and time to clinical outcomes, including the first use of use of medications (i.e., angiotensin-converting enzyme inhibitor [ACEi], angiotensin receptor blocker [ARB] or beta blocker), CKD progression, the incidence of kidney failure treated with KRT (i.e., initiation of chronic dialysis or kidney transplantation), first hospitalization for cardiovascular events, and all-cause mortality. We defined CKD progression as a sustained reduction in eGFR of more than 50%, sustained doubling of ACR or PCR, or a sustained increase (at least one level) in Udip measurements from baseline. We defined increases as “sustained” if all subsequent outpatient measurements (for eGFR, ACR, PCR, Udip) continued to meet the aforementioned criteria during the follow-up period. The incidence of kidney failure was identified using the Northern and Southern Alberta Renal Programs registries, as previously described [12]. We defined cardiovascular events as hospitalization for myocardial infarction, congestive heart failure, ischemic stroke or transient ischemic attack, percutaneous coronary intervention, or coronary artery bypass graft surgery. We identified all-cause mortality using provincial vital statistics.

### Statistical analysis

We described continuous variables using medians and interquartile ranges, and categorical variables as proportions. To compare differences between GD referrals and GC referrals, we used chi-square tests for categorical variables and Kruskal-Wallis tests for continuous variables. We used least squares regression analysis to identify the secular trend of the annual rate of nephrology and internal medicine visits from 2006 to 2019. To evaluate autocorrelations of the regression residuals, we used the C-H test/Dubin Wastin test [20, 21] and corrected any autocorrelations by incorporating Newey-West standard errors in the regression analysis [22]. We used multivariable Cox proportional hazard models to estimate the associations between GD and GC referrals and the outcomes of using renal-protective medication or a beta blocker, CKD progression, incident kidney failure, cardiovascular events, and all-cause mortality. For each outcome, if there were multiple events during follow-up (e.g., multiple ACEi prescriptions or multiple events of kidney function decline), we only considered the first event. The fully adjusted models included terms for age, sex, deprivation index, residential location, distance to nearest nephrology center, comorbid conditions, baseline kidney function (eGFR), presence of albuminuria, and hematuria. Participants were censored if they moved out of the province, reached the end of the study period (March 31, 2019), or died (except in the model for the outcome of death) were excluded from our analyses. We evaluated that the proportional hazard assumption was satisfied by examining plots of the log–negative log within-group survivorship functions versus log time. We analyzed the data using STATA version 17 and used *p* < 0.05 as the threshold for statistical significance.

## Results

### Participant characteristics

**Fig 1:**
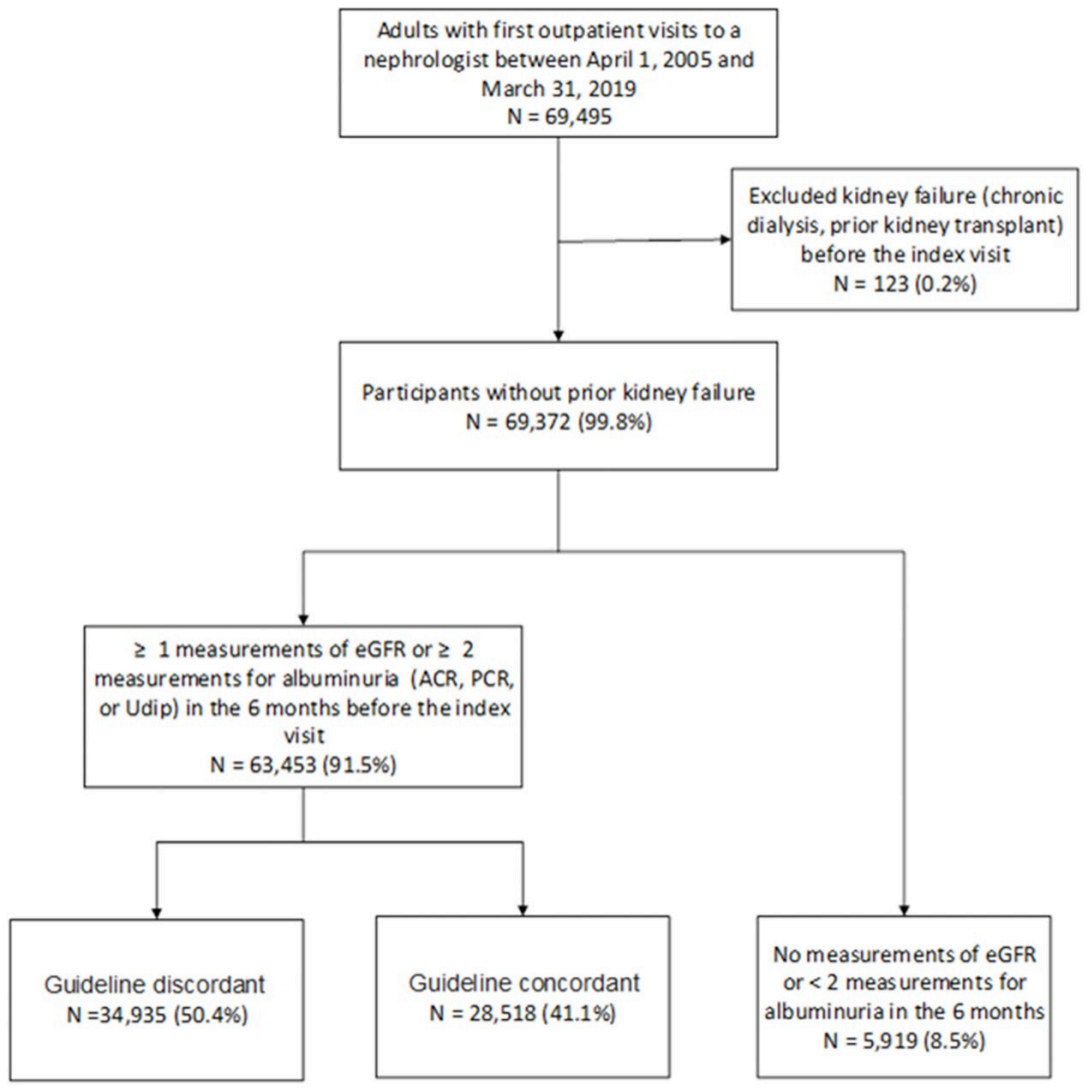
Flow chart: Cohort selection criteria. Outpatient eGFR and proteinuria measurements (ACR, PCR, or Udip) in the 6 months preceding the first nephrology visit were used to define guideline concordance of the referral. A visit was guideline concordant if any of the following criteria were satisfied: most recent eGFR < 30 mL/min per 1.73 m2, sustained proteinuria, or progressive decline in eGFR ≥ 5 mL/min per 1.73 m2. Proteinuria was defined as ACR ≥ 300 mg/g, PCR ≥ 500 mg/g, or Udip ≥ 2+, and sustained proteinuria was defined as at least two consecutive proteinuria measurements prior to the date of the nephrology visit. Decline in eGFR ≥ 5 mL/min per 1.73 m2 was defined as a decrease between the first and subsequent eGFR during the 6 months, and progressive decline was defined as at least two consecutive decreases in eGFR by ≥ 5 mL/min per 1.73 m2 prior to the date of nephology visit. Abbreviations: ACR, albumin-creatinine ratio; eGFR, estimated glomerular filtration rate; PCR: protein-creatinine ratio; Udip: urine dipstick.

Between April 1, 2005 and March 31, 2019, 69,495 adults 18 years of age or older residing in Alberta had initial outpatient visits to nephrologists (Fig 1). The study cohort included 69,372 (99.8%) patients who did not develop kidney failure before their visits. Of these participants, 34,935 (50%) had GD referrals, 28,518 (41%) had GC referrals, and 5,919 (9%) did not have an outpatient SCr measurement and/or had less than 2 urine protein measurements prior to the initial nephrology visit. Subjects with GD referrals were less likely to have completed hematuria measurements, and among those tested, were less likely to have hematuria (Table 1). Participants with GC referrals were more likely to be older; male; have a history of diabetes, hypertension, myocardial infarction, chronic heart failure, stroke or TIA, peripheral vascular disease, cancer, pulmonary disease, or dementia; and to have a higher deprivation index (Table 1).

**Table 1:** Patient baseline characteristics by appropriateness of nephrology referral.

Characteristic	All patients	Guideline discordant	Guideline concordant	No eGFR and albuminuria measurement ^a^	*p*-value
Number of patients (%)	69372	34935 (50.4)	28518 (41.1)	5919 (8.5)	
Age in years, median [IQR]	62.5 [47.8–74.3]	60.7 [46.1–72.5]	66.6 [53.5–77.3]	51.3 [36.4–65.8]	<0.001
< 40	11124 (16.0)	6088 (17.4)	3200 (11.2)	1836 (31.0)	<0.001
40–59	20108 (29.0)	10937 (31.3)	7101 (24.9)	2070 (35.0)	<0.001
60–79	28738 (41.4)	14275 (40.9)	12889 (45.2)	1574 (26.6)	<0.001
≥ 80	9402 (13.6)	3635 (10.4)	5328 (18.7)	439 (7.4)	<0.001
Gender, female (%)	35172 (50.7)	18485 (52.9)	13755 (48.2)	2932 (49.5)	<0.001
Urban location (%)	62245 (89.7)	31696 (90.7)	25449 (89.2)	5100 (86.2)	<0.001
Deprivation index^b^					
1 (least deprived)	11921 (17.2)	6309 (18.1)	4761 (16.7)	851 (14.4)	<0.001
2	11637 (16.8)	6028 (17.3)	4698 (16.5)	911 (15.4)	0.01
3	13094 (18.9)	6612 (18.9)	5379 (18.9)	1103 (18.6)	0.84
4	14927 (21.5)	7370 (21.1)	6231 (21.8)	1326 (22.4)	0.02
5 (most deprived)	16565 (23.9)	8049 (23.0)	6909 (24.2)	1607 (27.1)	<0.001
Distance to nearest nephrology center					
≤ 50 km	54594 (78.7)	28112 (80.5)	22120 (77.6)	4362 (73.7)	<0.001
50–100 km	5348 (7.7)	2436 (7.0)	2320 (8.1)	592 (10.0)	<0.001
> 100 km	9331 (13.5)	4348 (12.4)	4039 (14.2)	944 (15.9)	<0.001
Comorbid disease (%)					
Diabetes	23734 (34.2)	10070 (28.8)	12578 (44.1)	1086 (18.3)	<0.001
Hypertension	45839 (66.1)	21569 (61.7)	21736 (76.2)	2534 (42.8)	<0.001
Myocardial infarction	3698 (5.3)	1494 (4.3)	2022 (7.1)	182 (3.1)	<0.001
Chronic heart failure	8926 (12.9)	3320 (9.5)	5227 (18.3)	379 (6.4)	<0.001
Stroke or TIA	8468 (12.2)	3733 (10.7)	4284 (15.0)	451 (7.6)	<0.001
Peripheral vascular disease	2444 (3.5)	996 (2.9)	1341 (4.7)	107 (1.8)	<0.001
Cancer (lymphoma, metastatic, and non-metastatic)	4917 (7.1)	2425 (6.9)	2265 (7.9)	227 (3.8)	<0.001
Chronic pulmonary disease	12607 (18.2)	5664 (16.2)	6190 (21.7)	753 (12.7)	<0.001
Dementia	1973 (2.8)	751 (2.1)	1080 (3.8)	142 (2.4)	<0.001
Depression	4224 (6.1)	2242 (6.4)	1613 (5.7)	369 (6.2)	<0.001
Baseline eGFR in mL/min/1.73 m^2^, median [IQR]^c^	54.5 [36.7–85.7]	61.9 [43.3–92.2]	45.8 [27.4–73.1]	-	<0.001
eGFR category in mL/min/1.73m^2^					
≥60	27310 (39.4)	17455 (50.0)	9855 (34.6)	-	<0.001
45–59	11327 (16.3)	6785 (19.4)	4542 (15.9)	-	<0.001
30–44	14062 (20.3)	9401 (26.9)	4661 (16.3)	-	<0.001
15–29	8156 (11.8)	-	8156 (28.6)	-	-
<15	1066 (1.5)	-	1066 (3.7)	-	-
Progressive decline in eGFR ≥ 5 mL/min/1.73m^2^	11784 (17.0)	-	11784 (41.3)	-	-
Albuminuria present	12548 (18.1)	-	12548 (44.0)	-	-
Baseline ACR in mg/g^c^					
<30	13609 (19.6)	9130 (26.1)	4462 (15.6)	17 (0.3)	<0.001
30–300	9242 (13.3)	5669 (16.2)	3558 (12.5)	15 (0.3)	<0.001
>300	8195 (11.8)	755 (2.2)	7407 (26.0)	33 (0.6)	<0.001
Hematuria present	10141 (14.6)	4300 (12.3)	5805 (20.4)	36 (0.6)	<0.001

^a^ Includes patients with no eGFR measurements or less than 2 urine protein measurements, including albumin-creatinine ratio (ACR), protein-creatinine ratio (PCR), and urine dipstick (Udip).

^b^ An ecological measure of material deprivation expressed as a quintile (1 = least deprived to 5 = most deprived).

^c^ Baseline eGFR and ACR were defined as the most recent measurement before the initial nephrology visit. Median [IQR] eGFR was estimated for 33,641 patients whose referrals were guideline discordant, 28,280 patients whose referrals were guideline concordant, and the entire cohort of 61,921 patients.

*Notes*: Data are presented as number (%) except for age and eGFR, which are presented as median [interquartile range]. *p*-value is for the test comparing guideline concordant and guideline discordant referrals. Conversion factors for units: ACR in mg/g to mg/mmol, x 0.113.

*Abbreviations*: ACR: albumin-creatinine ratio; eGFR: estimated glomerular filtration rate; TIA: transient ischemic attack.

### Trend of nephrology referrals

**Fig 2:**
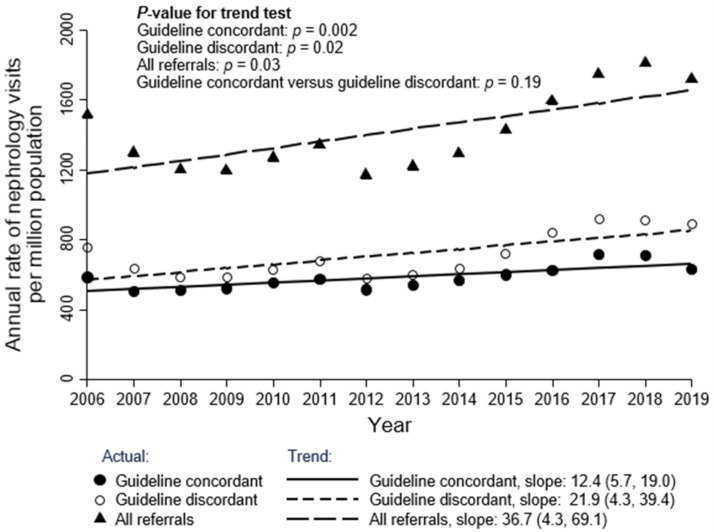
Annual rate of initial outpatient visits to nephrologists, by guideline concordant/discordant status. Each year corresponds to the period spanning from April 1 of the previous year to March 31 of that year (e.g., 2006 corresponds to April 1, 2005 to March 31, 2006).

**Fig 3:**
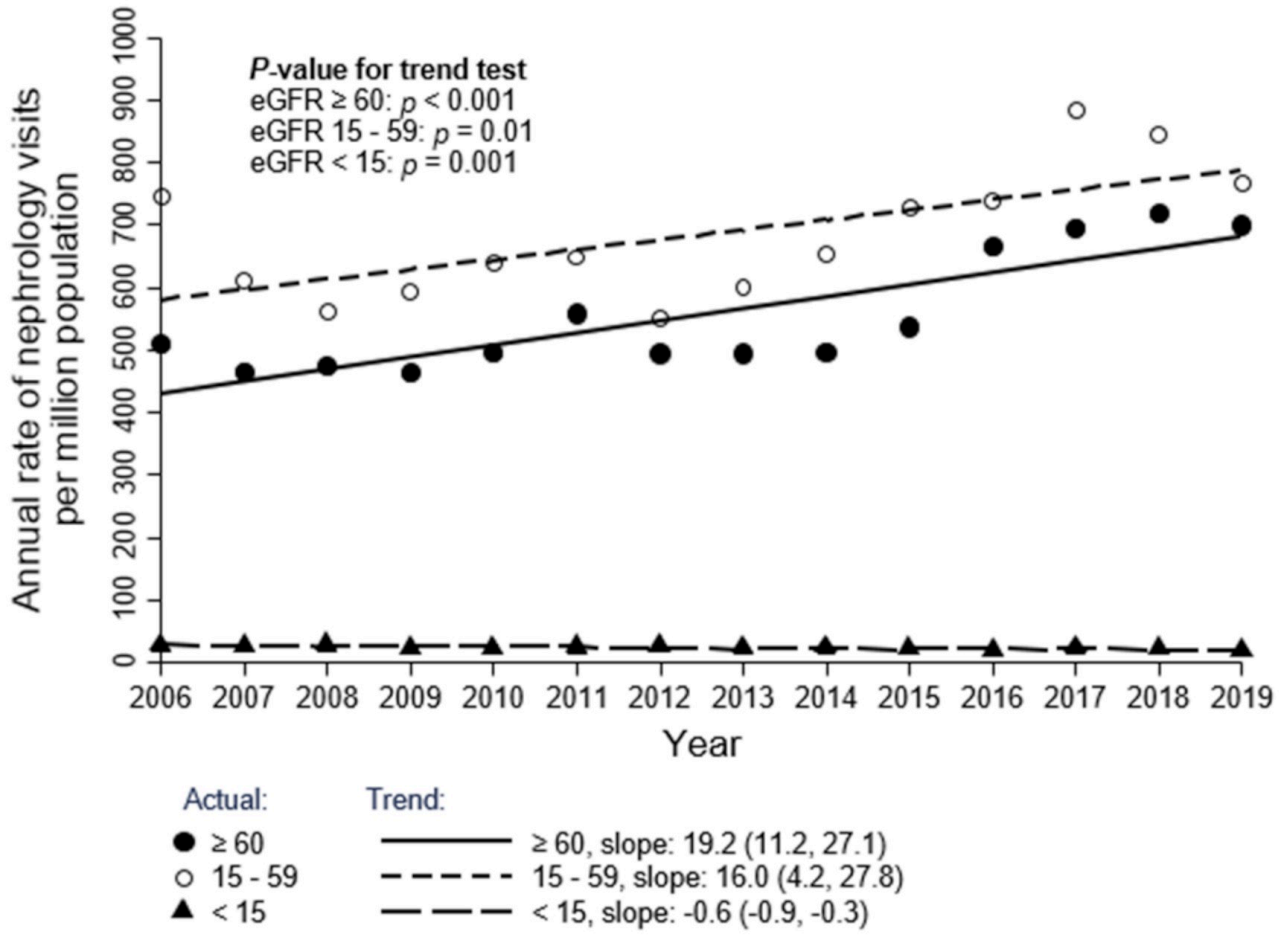
Annual rate of initial outpatient visits to nephrologists, by eGFR (ml/min per 1.73 m2) category. This cohort includes 61,921 patients who had at least one eGFR measurement in the 6 months prior to the initial nephrology visit. Each year corresponds to the period spanning from April 1 of the previous year to March 31 of that year (e.g., 2006 corresponds to April 1, 2005 to March 31, 2006).

**Fig 4:**
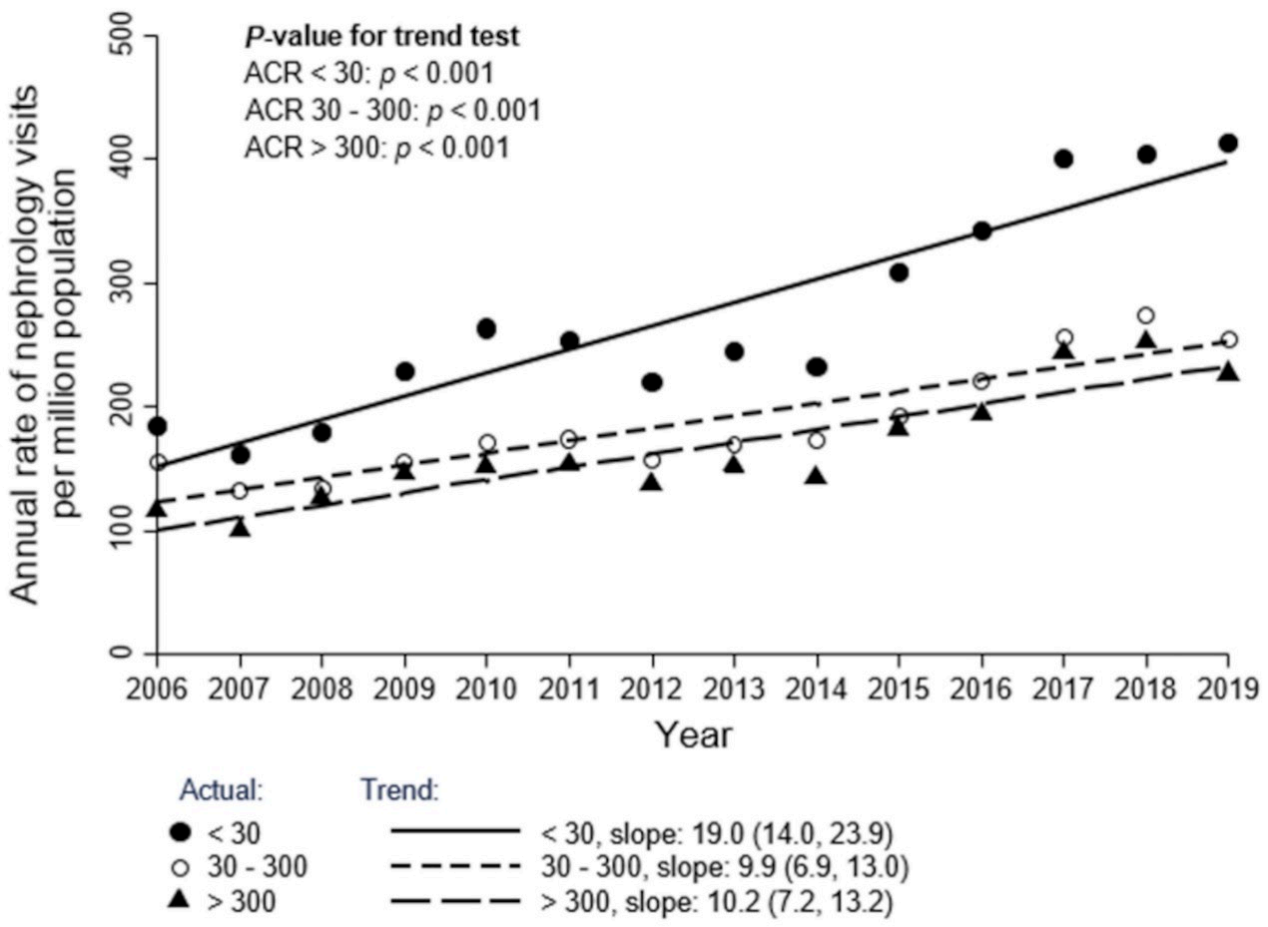
Annual rate of initial outpatient visits to nephrologists, by ACR (mg/g) category. This cohort includes 31,046 patients who had at least one ACR measurement in the 6 months prior to the initial nephrology visit. Each year corresponds to the period spanning from April 1 of the previous year to March 31 of that year (e.g., 2006 corresponds to April 1, 2005 to March 31, 2006). Conversion factors for units: ACR in mg/g to mg/mmol, x 0.113.

The overall rate of initial outpatient visits to nephrologists was 1513 pmp in 2006 and slightly increased each year to 1716 pmp in 2019 for all referrals (S1 Table in S1 File, Fig 2; trend: 36.7 pmp/year; 95% CI: 4.3, 69.1; *p* = 0.03). The overall rate of GC referrals was 584 pmp in 2006 and slightly increased each year to 629 pmp in 2019 (S1 Table in S1 File, Fig 2; trend: 12.4 pmp/year; 95% CI: 5.7, 19.0; *p* = 0.002). Both categories of referrals increased over time, with the overall rate of GD referrals increasing each year from 2006 to 2019 and to a greater extent over this period than GC referrals (S1 Table in S1 File, Fig 2; trend: 21.9 pmp/year; 95% CI: 4.3, 39.4; *p* = 0.02) although the difference is not statistically significant (*p* = 0.19). Patients with eGFR ≥ 60 ml/min/1.73 m^2^ (S2 Table in S1 File, Fig 3; trend: 19.2 pmp/year; 95% CI: 11.2, 27.1; *p* < 0.001) and ACR < 30 mg/g (S2 Table in S1 File, Fig 4; trend: 19.0 pmp/year; 95% CI: 14.0, 23.9; *p* < 0.001) had the highest referral rates and positive referral trends from 2006 to 2019 Age and sex standardized data show similar trends and are presented in the (S3-S6 Figs in S1 File).

### Trend of other internal medicine specialist visits

The overall rate of initial outpatient visits to internal medicine specialists for patients who also saw nephrologists was 387 pmp in 2006 and increased slightly each year to 860 pmp in 2019 (S3 Table, S1 Fig in S1 File; trend: 41.5 pmp/year; 95% CI: 31.6, 51.5; *p* < 0.001). The overall rate of increase for visits to internal medicine specialists for the GC group was 128 pmp in 2006 and rose to 286 pmp in 2019 (S3 Table, S1 Fig; in S1 File trend: 15.1 pmp/year; 95% CI: 12.5, 17.8; *p* < 0.001); for the GD group, this was 195 pmp in 2006 and rose to 461 pmp in 2019 (S3 Table, S1 Fig in S1 File; trend: 23.1 pmp/year; 95% CI: 17.4, 28.9; *p* < 0.001).

### Outcomes associated with guideline concordant vs. discordant referrals

Median longest follow-up time for time to event analysis was 4.4 years (IQR: 2.0–8.2 years). At baseline (1 year before the initial nephrology visit), patients in the GC group were more likely to be on ACEi, ARB, statin, or beta blocker medications (S5 Table in S1 File). After adjusting for age, sex, urban location, deprivation index, distance to nearest nephrology center, comorbid disease, and baseline hematuria, patients with GC referrals were more likely to be placed on ACEi/ARB or beta blockers (HR = 1.18; 95% CI: 1.15, 1.21; *p* < 0.001), experience CKD progression (HR = 1.09; 95% CI: 1.06, 1.13; *p* < 0.001), develop kidney failure (HR = 7.65; 95% CI: 6.83, 8.56; *p* < 0.001), have a cardiovascular event (HR = 1.40; 95% CI: 1.35,1.45; *p* < 0.001) or die (HR = 1.58; 95% CI: 1.52, 1.63; *p* < 0.001) compared to the GD cohort (Table 2, S2 Fig in S1 File). There were no differences between the GC and GD cohorts in use of SGLT2-inhibitors or GLP1 receptor agonists (S4 Table in S1 File).

**Table 2:** Associations between appropriateness of nephrology referral and clinical outcomes.

Appropriateness of referral	Use of ACEI/ARB or beta blocker^a^		CKD progression		Kidney failure		Cardiovascular events		Death	
	No. events/n	HR (95% CI)	No. events/n	HR (95% CI)	No. events/n	HR (95% CI)	No. events/n	HR (95% CI)	No. events/n	HR (95% CI)
Overall	36529/53906		13324/64356		2949/69372		16076/69372		13676/69372	
Guideline discordant	17040/27332	1[Referent]	6666/34935	1[Referent]	357/34935	1[Referent]	6335/34935	1[Referent]	4937/34935	1[Referent]
Guideline concordant	17655/22259	1.18 (1.15, 1.21)*	6583/28518	1.09 (1.06, 1.13)*	2388/28518	7.65 (6.83, 8.56)*	8842/28518	1.40 (1.35, 1.45)*	7928/28518	1.58 (1.52, 1.63)*
No eGFR or albuminuria measurements	1834/4315	0.95 (0.86, 1.05)	75/903	0.68 (0.54, 0.86)*	204/5919	3.35 (2.80, 4.00)*	899/5919	1.13 (1.05, 1.22)*	811/5919	1.24 (1.15, 1.34)*

*p < 0.05

^a^ To access the baseline use of ACEi, ARB and beta blocker medications, we only included patients whose initial nephrology visits occurred after January 1, 2009, as the PIN drug files date to January 1, 2008.

*Note*: Adjusted factors: age, sex, urban location, deprivation index, distance to nearest nephrology center, comorbid disease, and baseline hematuria. HRs for ACEi/ARB or beta blocker medications were also adjusted for baseline use. In the model for the outcome of CKD progression, only patients with at least one baseline eGFR or urine protein measurement were included.

*Abbreviations*: ACEi: angiotensin-converting enzyme inhibitor; ARB: angiotensin receptor blocker; CI: confidence interval; CKD: chronic kidney disease; eGFR: estimated glomerular filtration rate; HR: hazard ratio.

In our sub-group analyses, the likelihood of being placed on an ACEi/ARB or beta blocker was higher in patients with eGFR 15–59 mL/min/1.73m^2^ (HR = 1.06; 95% CI: 1.03, 1.09; *p* < 0.001) compared to those with eGFR ≥ 60 mL/min/1.73m^2^. This pattern was also seen in patients with albuminuria > 30 mg/g and patients over age 40, compared to those without albuminuria and those under age 40. Those with eGFR < 15 mL/min/1.73m^2^ were less likely to be prescribed ACEi/ARB or beta blocker (Table 3).

**Table 3:** Associations between baseline kidney function, age group, clinical outcomes.

Model	Use of ACEI/ARB or beta blocker^a^		CKD progression		Kidney failure		Cardiovascular events		Death	
	No. events/n	HR (95% CI)	No. events/n	HR (95% CI)	No. events/n	HR (95% CI)	No. events/n	HR (95% CI)	No. events/n	HR (95% CI)
Model 1: eGFR, mL/min/1.73 m^2^										
Overall	34211/48519		13074/61921		2711/61921		14974/61921		12657/61921	
≥ 60	11819/21629	1[Referent]	3986/27310	1[Referent]	289/27310	1[Referent]	3087/27310	1[Referent]	2257/27310	1[Referent]
15–59	21828/26120	1.06 (1.03, 1.09)*	8708/33545	1.61 (1.54, 1.68)*	2026/33545	9.41 (8.25, 10.73)*	11410/33545	1.36 (1.30, 1.43)*	9805/33545	1.40 (1.33, 1.47)*
<15	564/770	0.72 (0.66, 0.79)*	380/1066	4.32 (3.87, 4.82)*	396/1066	85.64 (72.77, 100.78)*	477/1066	1.79 (1.62, 1.98)*	595/1066	2.73 (2.48, 3.00)*
Model 2: ACR, mg/g										
Overall	19392/25761		8705/31046		1484/31046		7417/31046		5596/31046	
<30	7307/11433	1[Referent]	3680/13609	1[Referent]	94/13609	1[Referent]	2544/13609	1[Referent]	1834/13609	1[Referent]
30–300	6024/7562	1.19 (1.15, 1.24)*	3132/9242	1.22 (1.16, 1.28)*	230/9242	2.66 (2.08, 3.39)*	2316/9242	1.19 (1.12, 1.26)*	1830/9242	1.33 (1.24, 1.42)*
>300	6061/6766	1.41 (1.33, 1.50)*	1893/8195	1.12 (1.02, 1.23)*	1160/8195	9.54 (7.29, 12.49)*	2557/8195	1.53 (1.39, 1.68)*	1932/8195	1.65 (1.47, 1.84)*
Model 3: Age, year										
Overall	43458/53906		13324/64356		2949/69372		16076/69372		13676/69372	
<40	2876/8739	1[Referent]	1266/9551	1[Referent]	364/11124	1[Referent]	318/11124	1[Referent]	206/11124	1[Referent]
40–60	9145/15483	1.36 (1.31, 1.41)*	3217/18299	1.01 (0.95, 1.08)	1052/20108	0.96 (0.85, 1.09)	2532/20108	3.19 (2.83, 3.59)*	1661/20108	3.48 (3.00, 4.02)*
60–80	18324/22366	1.46 (1.40, 1.52)*	6722/27430	1.35 (1.26, 1.44)*	1371/28738	0.71 (0.62, 0.81)*	8864/28738	6.35 (5.65, 7.14)*	7110/28738	9.31 (8.06, 10.74)*
≥80	6718/7318	1.36 (1.30, 1.43)*	2119/9076	1.77 (1.64, 1.92)*	162/9402	0.31 (0.26, 0.38)*	4362/9402	10.15 (8.99, 11.45)*	4699/9402	22.06 (19.07, 25.54)*
Model 4: Appropriateness of referral										
Overall	4659/7392		1674/8537		302/9107		1702/9107		1380/9107	
Guideline discordant	2408/4184	1[Referent]	887/5158	1[Referent]	57/5158	1[Referent]	726/5158	1[Referent]	537/5158	1[Referent]
Guideline concordant	2014/2674	1.13 (1.05, 1.21)*	778/3241	1.23 (1.11, 1.36)*	230/3241	6.21 (4.62, 8.37)*	889/3241	1.40 (1.27, 1.55)*	773/3241	1.64 (1.46, 1.84)*
No eGFR or albuminuria measurements	237/534	1.13 (0.86, 1.49)	9/138	0.55 (0.28, 1.06)	15/708	1.49 (0.83, 2.68)	87/708	1.03 (0.82, 1.30)	70/708	1.10 (0.85, 1.42)

* *p* < 0.05.

^a^ To access the baseline use of ACEi, ARB and beta blocker medications, we only included patients whose index nephrology visits occurred after January 1, 2009, as the PIN drug files date to January 1, 2008.

*Note*: Conversion factors for units: ACR in mg/g to mg/mmol, x 0.113. All models adjusted for age, sex, urban location, deprivation index, distance to nearest nephrologist center, comorbid disease, and baseline hematuria. HRs for ACEi/ARB or beta blocker medications were also adjusted for baseline use, defined as any prescription filled within one year prior to the initial nephrology visit. In addition to these factors, Model 1 was adjusted for baseline albuminuria and included 61,921 patients with baseline eGFR measurements; Model 2 was adjusted for baseline eGFR categories and baseline albuminuria and included 31,046 patients with baseline ACR measurements; Model 4 included 9,107 subjects with a history of kidney stones or polycystic kidney disease.

*Abbreviations*: HR, hazard ratio; CI: confidence interval; No, number; ACEI, angiotensin-converting enzyme inhibitors; ARB, angiotensin receptor blocker; CKD, chronic kidney disease; eGFR, estimated glomerular filtration rate; ACR, albumin-creatinine ratio.

Overall, patients with lower eGFR and higher albuminuria were at increased risk for CKD progression, kidney failure, cardiovascular events, and death. Kidney failure was less likely for older patients (age > 80 years: HR = 0.31; 95% CI: 0.26, 0.38; *p* < 0.001; age 60–80 years: HR = 0.71; 95% CI: 0.62, 0.81; *p* < 0.001) than for patients younger than 40.

## Discussion

In this retrospective cohort study of 69,372 patients, we found that between 2006 and 2019, the overall trend in referrals to nephrologists increased, with only 41% being GC. The absolute value of the trend for the increase in referrals was greatest for the GD group (although not statistically significant) and for patients with eGFR ≥ 60 ml/min/1.73 m^2^ and ACR < 30 mg/g. Overall, this suggests that many of the referred patients being seen by nephrologists have relatively mild CKD and may be more suitable for continued PCP surveillance, as per guideline recommendations. Of note, although the term “guideline discordant” may also apply to late referrals, we designed our analysis to focus only on early GD referrals, as this is the lesser-studied entity. We also found an increasing overall trend to other internal medicine specialists which likely reflects an increasing trend of chronic diseases and multi-morbidity in Alberta, Canada [23].

Prior studies have shown that the introduction of eGFR reporting was associated with an increase in the number of nephrology referrals [24–27]. Evidence regarding whether these referrals were GD was mixed, with Akbari et al. showing no change [27], Noble et al. reporting a slight reduction in GC referrals [26], and Hingwala et al. showing a consistently high rate (62.7%) of GD referrals [28]. Our study period begins in 2004, when eGFR began to be universally reported by laboratories in Alberta. Prior studies have shown various rates of GD referrals, with Hommel et al. showing 25% [7], Wright et al. showing 40% [6], and Hingwala et al. showing that 62.7% [28] of referrals to nephrologists are GD. These studies however were limited by short follow-up times and relatively small sample sizes. Our study adds to existing knowledge by providing a large population-based analysis with 13 years of follow-up time.

There may be several reasons for the high proportion of GD referrals. First, PCPs and nephrologists may have different interpretations of each other’s responsibilities. For PCPs, the focus of collaborative care with nephrologists is on slowing CKD progression and managing hypertension and diabetes, which typically are priorities in early CKD. In contrast, the focus of collaborative care for nephrologists tends to be on preparation for KRT [29]. Second, the expanding number and complexity of guidelines increases the burden on PCPs [30–32]. This burden is amplified by known challenges with interprofessional communication between PCPs and specialists, as well as unclear division of responsibilities in the co-management of CKD patients [33, 34]. Third, there are challenges associated with PCPs managing CKD. Despite PCPs having a significant and expanding role in CKD management, several domains of CKD care may be enhanced, with follow-up urine ACR being the quality indicator most significantly lacking [32].

Patients with GC referrals more often had vascular disease, diabetes, hypertension, pulmonary disease, dementia, and cancer. These specific comorbidities are associated with higher rates of death and hospitalization in the CKD population [35]. The GC cohort also was older, and patients over age 60 were at significantly higher risk for death and cardiovascular events. Thus, we attribute the higher risk of cardiovascular events and death in the GC group to higher rates of comorbidities and advanced age, rather than to late referrals per se.

Our study shows a need to enhance current methods for referring CKD patients to nephrology. Prolonged waiting times in the Canadian healthcare system limit timely access to specialist care [36]. A significant number of referrals have been shown to be missing necessary information, making it difficult to triage patients and determine the appropriateness of referrals [6] In a meta-analysis, Akbari et al [37] highlighted several interventions to improve outpatient referral appropriateness. Two options include using structured referral forms and involving consultants in education activities, although neither improved the appropriateness of referrals from primary care in a CKD cohort [38].

Centralized systems for managing CKD referrals have been shown to reduce wait times significantly in Canadian cohorts [39, 40]. Use of renal care pathways such as the Alberta Kidney Pathway [41] have made referral and triage processes more efficient [42]. The Alberta Kidney Pathway has shown to lead to an increase in urine ACR testing especially in non-diabetic patients [43] and prior work has examined ways to disseminate this tool locally [44]. In studies focused on electronic advice requests and consults to nephrologists where 18% [45] to 27% [46] of the consults were related to CKD, researchers found that only a minority of electronic system referrals led to in-person consults [45, 46] highlighting the potential of e-referrals to reduce in-person nephrology visits. Future work thus needs to be directed toward empowering PCPs and providing convenient access to CKD management guidelines to identify interventions that can enhance CKD referral patterns. Our group is currently conducting a systematic review examining various published quality improvement (QI) studies aimed at enhancing referral patterns for CKD patients to nephrology [47]. We hope that from this review, we can identify effective QI initiatives to implement in our province to increase the proportion of referrals to nephrology that are GC.

This study should be interpreted considering its limitations. First, we did not include patients exclusively referred for reasons not based on eGFR and/or proteinuria measures, such as recurrent nephrolithiasis, refractory hypertension, and cystic kidney diseases [3], as there is a lack of validated methods for identifying such patients from administrative data. Furthermore, it is unclear whether PCPs in Alberta are more likely to rely on Canadian Society of Nephrology (CSN) recommendations [48] or to adhere to KDIGO guidelines [3] when referring CKD patients to nephrology. CSN recommends referral for UACR > 60 mg/mmol which is based on the likelihood of patients with this degree of albuminuria or proteinuria > 1g/day needing immunosuppressive medications and/or biopsy [48]. Thus, the number of GD referrals in our analysis would have been higher if we had utilized the CSN recommendations. Of note, it is also not clear how familiar PCPs in Alberta are with either CSN or KDIGO guidelines at the time of referral particularly over the previous decade, and further work is needed to gauge the awareness of these guidelines in the primary care community.

We also acknowledge that there may be reasons for referrals which are not explicitly covered in the guidelines but involve practices which may be outside the comfort zone of a PCP. These include initiation of SGLT-2 inhibitors or dealing with polypharmacy in a patient with impaired renal function but eGFR > 30. However, it was not feasible to ascertain all reasons for referrals within our large cohort.

In conclusion, a large proportion of referrals to nephrology do not appear to meet the criteria stipulated in current practice guideline recommendations. Our study highlights the need for quality improvement initiatives to enhance referral patterns of patients with CKD to increase appropriateness and efficiency in the delivery of specialist kidney care and to support PCPs in early CKD management.

## Supporting information

S1 File Contains all supporting tables and figures. (DOCX)
