# Supplementary material for: Trends in nephrology referral patterns for patients with chronic kidney disease: Retrospective cohort study
Source: PLoS One. 2022 Aug 11;17(8):e0272689. doi: 10.1371/journal.pone.0272689 (PMC9371302; doi:10.1371/journal.pone.0272689)
Supplement: S1 File — (DOCX) [file pone.0272689.s001.docx]

Supplementary files

[Supplementary Table 1. Annual rate of nephrology referrals from 2006 to 2019, pmp (number of initial outpatient visits to nephrologists per million population) 2](#_Toc103269338)

[Supplementary Table 2. Annual rate of nephrology referrals from 2006 to 2019 by eGFR and ACR categories, pmp (number of initial outpatient visits to nephrologists per million population) 3](#_Toc103269339)

[Supplementary Table 3. Annual rate of initial outpatient visits to other internal medicine specialists from 2006 to 2019, pmp (number of initial outpatient visits to other internal medicine specialists per million population) 4](#_Toc103269340)

[Supplementary Table 4: Associations between patient characteristics and the use of SGLT2i, GLP1, and statins 5](#_Toc103269341)

[Supplementary Table 5. Patient characteristics and use of appropriate medications at baseline 7](#_Toc103269342)

[Supplementary Figure 1. Annual rate for patients who had both internal medicine and nephrology visits (n = 30707) 8](#_Toc103269343)

[Supplementary Figure 2: Associations between clinical outcomes and referrals to nephrologists 9](#_Toc103269344)

[Supplementary Figure 3. Age and sex standardized annual rate of initial outpatient visits to nephrologists, by guideline concordant/discordant status 10](#_Toc103269345)

[Supplementary Figure 4. Age and sex standardized annual rate of initial outpatient visits to nephrologists, by eGFR (ml/min per 1.73 m^2^) category. 11](#_Toc103269346)

[Supplementary Figure 5. Age and sex standardized annual rate of initial outpatient visits to nephrologists, by ACR (mg/g) category 12](#_Toc103269347)

[Supplementary Figure 6. Age and sex standardized annual rate for patients who had both internal medicine and nephrology visits (n = 30707) 13](#_Toc103269348)

# Supplementary Table 1. Annual rate of nephrology referrals from 2006 to 2019, pmp (number of initial outpatient visits to nephrologists per million population)

| Year | All referrals | Guideline concordant | Guideline discordant | No eGFR or proteinuria measurement |
| --- | --- | --- | --- | --- |
| 2006 | 1513 | 584 | 756 | 173 |
| 2007 | 1295 | 505 | 634 | 156 |
| 2008 | 1200 | 509 | 581 | 110 |
| 2009 | 1195 | 520 | 581 | 94 |
| 2010 | 1266 | 553 | 624 | 88 |
| 2011 | 1341 | 571 | 679 | 91 |
| 2012 | 1167 | 513 | 577 | 77 |
| 2013 | 1216 | 540 | 602 | 74 |
| 2014 | 1292 | 565 | 636 | 90 |
| 2015 | 1425 | 598 | 718 | 109 |
| 2016 | 1592 | 625 | 842 | 126 |
| 2017 | 1747 | 714 | 917 | 116 |
| 2018 | 1809 | 708 | 911 | 190 |
| 2019 | 1716 | 629 | 886 | 200 |

*Note:* Each year corresponds to the period spanning from April 1of the previous year to March 31 of that year (e.g., 2006 corresponds to April 1, 2005 to March 31, 2006).

# Supplementary Table 2. Annual rate of nephrology referrals from 2006 to 2019 by eGFR and ACR categories, pmp (number of initial outpatient visits to nephrologists per million population)

| Year | eGFR, ml/min per 1.73 m^2^ | | | | ACR, mg/g | | | | Both eGFR and ACR | |
| --- | --- | --- | --- | --- | --- | --- | --- | --- | --- | --- |
|  | All | ≥ 60 | 15-59 | <15 | All | <30 | 30-300 | >300 |  |  |
| 2006 | 1280 | 509 | 745 | 26 | 453 | 184 | 154 | 115 | 434 |  |
| 2007 | 1100 | 464 | 611 | 25 | 392 | 161 | 132 | 99 | 378 |  |
| 2008 | 1061 | 474 | 560 | 27 | 439 | 179 | 134 | 126 | 429 |  |
| 2009 | 1078 | 463 | 594 | 22 | 528 | 228 | 155 | 145 | 518 |  |
| 2010 | 1157 | 495 | 641 | 21 | 585 | 263 | 171 | 151 | 576 |  |
| 2011 | 1230 | 558 | 649 | 23 | 580 | 253 | 174 | 153 | 574 |  |
| 2012 | 1071 | 495 | 552 | 24 | 514 | 220 | 157 | 137 | 507 |  |
| 2013 | 1117 | 495 | 600 | 22 | 565 | 245 | 169 | 151 | 558 |  |
| 2014 | 1175 | 497 | 655 | 23 | 545 | 232 | 172 | 141 | 537 |  |
| 2015 | 1284 | 536 | 727 | 20 | 682 | 309 | 192 | 182 | 670 |  |
| 2016 | 1420 | 665 | 738 | 17 | 756 | 342 | 221 | 194 | 738 |  |
| 2017 | 1601 | 696 | 883 | 23 | 899 | 400 | 256 | 243 | 888 |  |
| 2018 | 1583 | 718 | 844 | 20 | 929 | 404 | 274 | 251 | 917 |  |
| 2019 | 1485 | 700 | 768 | 16 | 893 | 414 | 254 | 226 | 882 |  |

*Note:* Each year corresponds to the period spanning from April 1of the previous year to March 31 of that year (e.g., 2006 corresponds to April 1, 2005 to March 31, 2006).

Abbreviations: ACR: albumin-creatinine ratio; eGFR: estimated glomerular filtration rate.

# Supplementary Table 3. Annual rate of initial outpatient visits to other internal medicine specialists from 2006 to 2019, pmp (number of initial outpatient visits to other internal medicine specialists per million population)

| Year | All internal medicine visits | Patients with both nephrology visits and internal medicine visits^a^ | | | |
| --- | --- | --- | --- | --- | --- |
|  |  | All | Guideline concordant | Guideline discordant | No eGFR or albuminuria measurement |
| 2006 | 22489 | 387 | 128 | 195 | 64 |
| 2007 | 26160 | 364 | 123 | 186 | 55 |
| 2008 | 27844 | 377 | 139 | 191 | 47 |
| 2009 | 28513 | 437 | 180 | 211 | 46 |
| 2010 | 29269 | 454 | 184 | 230 | 40 |
| 2011 | 25807 | 514 | 202 | 271 | 41 |
| 2012 | 27801 | 489 | 202 | 247 | 40 |
| 2013 | 28104 | 481 | 197 | 250 | 35 |
| 2014 | 30580 | 544 | 228 | 271 | 45 |
| 2015 | 31004 | 641 | 254 | 332 | 55 |
| 2016 | 29850 | 740 | 272 | 404 | 64 |
| 2017 | 27769 | 812 | 318 | 435 | 58 |
| 2018 | 25467 | 881 | 319 | 460 | 102 |
| 2019 | 24348 | 860 | 286 | 461 | 113 |

^a^ Includes patients who visited both nephrologists and other internal medicine specialists from 2006 to 2019. Internal medicine includes general internal medicine, cardiology, and endocrinology.

*Note:* Each year corresponds to the period spanning from April 1 of the previous year to March 31 of that year (e.g., 2006 corresponds to April 1, 2005 to March 31, 2006).

# Supplementary Table 4: Associations between patient characteristics and the use of SGLT2i, GLP1, and statins

| Patient characteristic | Use of SGLT2i | | Use of GLP1 | | Use of statins | |
| --- | --- | --- | --- | --- | --- | --- |
|  | No. events/n | HR (95% CI) | No. events/n | HR (95% CI) | No. events/n | HR (95% CI) |
| Appropriateness of referral |  |  |  |  |  |  |
| Overall | 1014/25879 |  | 921/44292 |  | 27180/53906 |  |
| Guideline discordant | 510/13404 | 1[Referent] | 504/22551 | 1[Referent] | 12709/27332 | 1[Referent] |
| Guideline concordant | 462/10091 | 1.06 (0.93, 1.21) | 381/18088 | 0.98 (0.85, 1.13) | 13246/22259 | 1.10 (1.07, 1.13)* |
| No eGFR or albuminuria | 42/2384 | 1.04 (0.58, 1.86) | 36/3653 | 0.64 (0.38, 1.10) | 1225/4315 | 1.12 (0.99, 1.27) |
|  |  |  |  |  |  |  |
| eGFR, mL/min/1.73 m2 |  |  |  |  |  |  |
| Overall | 956/22954 |  | 861/39723 |  | 27180/48519 |  |
| ≥ 60 | 552/10481 | 1[Referent] | 481/17774 | 1[Referent] | 8039/21629 | 1[Referent] |
| 15–59 | 404/12187 | 0.50 (0.43, 0.58)* | 376/21343 | 0.74 (0.64, 0.87)* | 17198/26120 | 1.19 (1.15, 1.23)* |
| < 15 | 0/286 | - | 4/606 | 0.32 (0.12, 0.88)* | 413/770 | 0.83 (0.75, 0.91)* |
|  |  |  |  |  |  |  |
| ACR, mg/mmol |  |  |  |  |  |  |
| Overall | 783/13111 |  | 657/21443 |  | 15419/25761 |  |
| <3 | 242/5880 | 1[Referent] | 231/9513 | 1[Referent] | 5917/11433 | 1[Referent] |
| 3–30 | 238/3786 | 0.98 (0.82, 1.18) | 208/6279 | 0.99 (0.82, 1.20) | 4730/7562 | 1.05 (1.01, 1.09)* |
| >30 | 303/3445 | 0.95 (0.70, 1.30) | 218/5651 | 1.18 (0.86, 1.63) | 4772/6766 | 1.13 (1.05, 1.21)* |
|  |  |  |  |  |  |  |
| Age |  |  |  |  |  |  |
| Overall | 1014/25879 |  | 921/44292 |  | 27180/53906 |  |
| <40 | 62/4285 | 1[Referent] | 110/7173 | 1[Referent] | 933/8739 | 1[Referent] |
| 40–60 | 382/7216 | 1.01 (0.76, 1.34) | 446/12666 | 0.74 (0.59, 0.92)* | 6731/15483 | 2.56 (2.39, 2.75)* |
| 60–80 | 540/10945 | 0.77 (0.58, 1.03) | 356/18385 | 0.42 (0.33, 0.54)* | 15240/22366 | 2.74 (2.55, 2.95)* |
| ≥ 80 | 30/3433 | 0.29 (0.18, 0.46)* | 9/6068 | 0.06 (0.03, 0.13)* | 4276/7318 | 2.50 (2.31, 2.70)* |

* *p* < 0.05

*Notes:* PIN medication data are available from January 1, 2008, but files for specific medications begin on different dates: January 1, 2008 (statins), May 2010 (GLP1), and May 2014 (SGLT2i). Thus, we include patients whose initial nephrology visits occurred after January 1, 2009 (statins), April 1, 2011 (GLP1), and April 1, 2015 (SGLT2i) to access their baseline use of these medications. Adjusted factors: age, sex, urban location, deprivation index, distance to nearest nephrology center, comorbid disease, and baseline hematuria. HRs for SGLT2i, GLP1 and statins were also adjusted for baseline use of the mediations, defined as any prescription within 1 year prior to the initial nephrology visit. In the model for CKD progression, only patients with at least one baseline eGFR or urine protein measurement were included.

*Abbreviations:* ACR: albumin-creatinine ratio; CI: confidence interval; CKD: chronic kidney disease; eGFR: estimated glomerular filtration rate; GLP1: glucagon-like peptide-1; HR: hazard ratio; SGLT2i: sodium-glucose co-transporter 2 inhibitor.

# Supplementary Table 5. Patient characteristics and use of appropriate medications at baseline

| Baseline characteristic | All patients  N (%) | Guideline discordant  N (%) | Guideline concordant  N (%) | No eGFR or albuminuria measurement  N (%) | *p*-value |
| --- | --- | --- | --- | --- | --- |
| Medication use | |  |  |  |  |
| Cohort A | 53906 (100.0) | 27332 (50.7) | 22259 (41.3) | 4315 (8.0) |  |
| Statin | 21334 (39.6) | 9870 (36.1) | 10677 (48.0) | 787 (18.2) | <0.001 |
| ACEi | 16739 (31.1) | 7638 (27.9) | 8438 (37.9) | 663 (15.4) | <0.001 |
| ARB | 15071 (28.0) | 6890 (25.2) | 7652 (34.4) | 529 (12.3) | <0.001 |
| Beta blocker | 14310 (26.5) | 6218 (22.7) | 7528 (33.8) | 564 (13.1) | <0.001 |
|  |  |  |  |  |  |
| Cohort B | 44292 (100.0) | 22551 (50.9) | 18088 (40.8) | 3653 (8.2) |  |
| GLP1 | 417 (0.9) | 215 (1.0) | 290 (2.9) | 19 (0.8) | 0.302 |
|  |  |  |  |  |  |
| Cohort C | 25879 (100.0) | 13404 (51.8) | 10091 (39.0) | 2384 (9.2) |  |
| SGLT2i | 598 (2.3) | 289 (2.2) | 290 (2.9) | 19 (0.8) | <0.001 |
|  |  |  |  |  |  |
| Previous measurements |  |  |  |  |  |
| Cohort D | 22483 (100.0) | 14474 (64.4) | 8009 (35.6) | - |  |
| eGFR≥90, and ACR<30, and no hematuria | 2648 (11.9) | 2025 (18.4) | 623 (5.5) | - | <0.001 |
| eGFR≥60, and ACR<30, and no hematuria | 5593 (25.1) | 3963 (36.0) | 1630 (14.5) | - | <0.001 |

*Notes:* Baseline medication is defined as at least one prescription in the year prior to the initial nephrology visit. PIN medication data are available from January 1, 2008, but files for specific medications begin on different dates: January 1, 2008 (Cohort A), May 2010 (Cohort B), and May 2014 (Cohort C). Thus, we include patients whose initial nephrology visits occurred after January 1, 2009 (Cohort A), April 1, 2011 (Cohort B), and April 1, 2015 (Cohort C). To assess previous measurements, we included patients with at least one eGFR, one ACR, and one urine red blood cell measurement (Cohort D).

*Abbreviations:* ACEi: angiotensin-converting enzyme inhibitor; ACR, albumin-creatinine ratio; ARB: angiotensin receptor blocker; GLP1: glucagon-like peptide-1; eGFR, estimated glomerular filtration rate; SGLT2i: sodium-glucose co-transporter 2 inhibitor.

# Supplementary Figure 1. Annual rate for patients who had both internal medicine and nephrology visits (n = 30707)

# Supplementary Figure 2: Associations between clinical outcomes and referrals to nephrologists

Overall

Guideline discordant

Guideline concordant

No eGFR and albuminuria

measurements

Use of ACEI/ARB or Beta blocker

36529/53906

17040/27332

1.00 [Referent]

17655/22259

1.18 (1.15, 1.21)

1834/4315

0.95 (0.86, 1.05)

Overall

Guideline discordant

Guideline concordant

No eGFR and albuminuria

measurements

CKD progression

13324/64356

6666/34935

1.00 [Referent]

6583/28518

1.09 (1.06, 1.13)

75/903

0.68 (0.54, 0.86)

Overall

Guideline discordant

Guideline concordant

No eGFR and albuminuria

measurements

Kidney failure

2949/69372

357/34935

1.00 [Referent]

2388/28518

7.65 (6.83, 8.56)

204/5919

3.35 (2.80, 4.00)

Overall

Guideline discordant

Guideline concordant

No eGFR and albuminuria

measurements

Cardiovascular events

16076/69372

6335/34935

1.00 [Referent]

8842/28518

1.40 (1.35, 1.45)

899/5919

1.13 (1.05, 1.22)

Overall

Guideline discordant

Guideline concordant

No eGFR and albuminuria

measurements

Death

13676/69372

4937/34935

1.00 [Referent]

7928/28518

1.58 (1.52, 1.63)

811/5919

1.24 (1.15, 1.34)

0.5

1

2

5

10

0.5

1

2

5

10

Guideline concordant/discordant

No. event/N

HR (95% CI)

HR (95% CI)

*Notes:* Adjusted factors: age, sex, urban location, deprivation index, distance to nearest nephrologist center, comorbid disease, and baseline hematuria. HRs for ACEi/ARB or beta blocker medications were also adjusted for baseline use. In the model for CKD progression, only patients with at least one baseline eGFR or urine protein measurement were included.

*Abbreviations:* ACEi: angiotensin-converting enzyme inhibitor; ARB: angiotensin receptor blocker; CI: confidence interval; CKD: chronic kidney disease; eGFR: estimated glomerular filtration rate; HR: hazard ratio.

# Supplementary Figure 3. Age and sex standardized annual rate of initial outpatient visits to nephrologists, by guideline concordant/discordant status

*Note:* Each year corresponds to the period from April 1 of the previous year to March 31 of that year (e.g., 2006 corresponds to April 1, 2005 to March 31, 2006).

# Supplementary Figure 4. Age and sex standardized annual rate of initial outpatient visits to nephrologists, by eGFR (ml/min per 1.73 m^2^) category.

# Supplementary Figure 5. Age and sex standardized annual rate of initial outpatient visits to nephrologists, by ACR (mg/g) category

# Supplementary Figure 6. Age and sex standardized annual rate for patients who had both internal medicine and nephrology visits (n = 30707)
